# Maternal health surveillance panel: a tool for expanding epidemiological surveillance of women’s health and its determinants

**DOI:** 10.1590/1980-549720240009

**Published:** 2024-02-26

**Authors:** Rosa Maria Soares Madeira Domingues, Agatha Sacramento Rodrigues, Marcos Augusto Bastos Dias, Valeria Saraceni, Rossana Pulcineli Vieira Francisco, Rejane Sobrinho Pinheiro, Claudia Medina Coeli

**Affiliations:** I Instituto Nacional de Infectologia Evandro Chagas - Rio de Janeiro (RJ), Brazil; II Universidade Federal do Espírito Santo - Vitória (ES), Brazil.; III Instituto Nacional de Saúde da Mulher, da Criança e do Adolescente Fernandes Figueira - Rio de Janeiro (RJ), Brazil.; IV Secretaria Municipal de Saúde do Rio de Janeiro - Rio de Janeiro (RJ), Brazil.; V Universidade de São Paulo - São Paulo (SP), Brazil.; VI Universidade Federal do Rio de Janeiro, Instituto de Estudos em Saúde Coletiva - Rio de Janeiro (RJ), Brazil.

**Keywords:** Epidemiological monitoring, Maternal health, Maternal death, Information systems, Vigilância epidemiológica, Saúde materna, Morte materna, Sistemas de informação

## Abstract

**Objective:**

To present the methodology used in the development of two products for maternal health surveillance and its determinants and discuss their possible uses.

**Methods:**

Based on a theoretical model of the determinants of maternal death and databases of Brazilian health information systems, two free products were developed: an interactive panel “surveillance of maternal health” and an educational material “Aparecida: a story about the vulnerability of Brazilian women to maternal death”, both available on the website of the Brazilian Obstetric Observatory.

**Results:**

More than 30 indicators were calculated for the period 2012-2020, containing information on socioeconomic conditions and access to health services, reproductive planning, prenatal care, delivery care, conditions of birth and maternal mortality and morbidity. The indicators related to severe maternal morbidity in public hospitalizations stand out, calculated for the first time for the country. The panel allows analysis by municipality or aggregated by health region, state, macro-region and country; historical series analysis; and comparisons across locations and with benchmarks. Information quality data are presented and discussed in an integrated manner with the indicators. In the educational material, visualizations with national and international data are presented, aiming to help in the understanding of the determinants of maternal death and facilitate the interpretation of the indicators.

**Conclusion:**

It is expected that the two products have the potential to expand epidemiological surveillance of maternal health and its determinants, contributing to the formulation of health policies and actions that promote women’s health and reduce maternal mortality.

## RESUME

**Objetivo:**

Apresentar a metodologia utilizada no desenvolvimento de dois produtos para a vigilância da saúde materna e seus determinantes e discutir as suas possíveis utilizações.

**Métodos:**

A partir de modelo teórico dos determinantes do óbito materno e bases de dados dos sistemas de informação em saúde brasileiros, foram desenvolvidos dois produtos gratuitos: um painel interativo denominado “Vigilância da saúde materna” e um material educativo chamado “Aparecida: uma história sobre a vulnerabilidade da mulher brasileira à morte materna”, ambos disponíveis no site do Observatório Obstétrico Brasileiro.

**Resultados:**

Foram calculados mais de 30 indicadores para o período 2012-2020, contendo informações sobre condições socioeconômicas e de acesso a serviços de saúde, planejamento reprodutivo, assistência pré-natal, assistência ao parto, condições de nascimento e mortalidade e morbidade materna. Destacam-se os indicadores relacionados à morbidade materna grave em internações públicas, calculados pela primeira vez para o país. O painel permite análises por município ou agregadas por região de saúde, unidade da federação, macrorregião e país; análises de série histórica; e comparações entre localidades e com padrões de referência. Dados de qualidade da informação são apresentados e discutidos de forma integrada aos indicadores. No material educativo, visualizações com dados nacionais e internacionais são apresentadas, visando auxiliar na compreensão dos determinantes do óbito materno e facilitar a interpretação dos indicadores.

**Conclusão:**

Espera-se que os produtos tenham o potencial de ampliar a vigilância epidemiológica da saúde materna e seus determinantes, contribuindo para a formulação de políticas e ações de saúde que promovam a saúde das mulheres e reduzam a mortalidade materna.

## INTRODUCTION

Maternal death is a major public health problem in Brazil and worldwide. Globally, Maternal Mortality Ratio (MMR) was estimated to be 223 per 100,000 live births (LB) in 2020, with higher values in low- and middle-income countries and stagnation of the downward trend between 2016 and 2020^1^ ^,^ ^2^.

In Brazil, MMR has shown a downward trend since the 1990s^3^, with a less pronounced decline from the year 2000 onward, but still much higher than the targets of the Millennium Development Goals and the Sustainable Development Goals, agreed internationally^4^.

Despite the high MMR values, maternal death is an infrequent event, especially in the context of health services or in places with a small number of births. In the 2012-2020 period, 43% of Brazilian municipalities recorded no occurrences of maternal death^5^, limiting the usefulness of investigation these deaths for formulating strategies to promote maternal health in these contexts.

Maternal mortality has multiple determinants^6^, being affected by the social condition of women, access to reproductive health and prenatal care (PN) and childbirth services, and by clinical and obstetric complications that may occur during pregnancy, childbirth, and the postpartum period. Considering this context, the research question is: “Is it possible to use other social and health indicators to expand maternal health surveillance with a view to preventing and controlling maternal deaths?”

As part of a call for data science in women’s health, two products were developed to expand maternal health surveillance and knowledge about the determinants of maternal death. The objective of this article was to present the methodology adopted in the development of these products and discuss their possible uses.

### Steps in product development

Two products were developed, with free access, hosted on the Brazilian Obstetric Observatory website: the panel “Maternal health surveillance” (https://observatorioobstetrico.shinyapps.io/painel-vigilancia-saude-materna/) and “Aparecida: a story about the vulnerability of Brazilian women to maternal death” (https://observatorioobstetricobr.org/a-historia-de-aparecida/). Both products were developed with de-identified data from Brazilian information systems publicly available on the website of the Information Technology Department of the Unified Health System (*Departamento de Informática do Sistema Único de Saúde* - DATASUS): Live Birth Information System (*Sistema de Informação sobre Nascidos Vivos* - SINASC), Mortality Information System (*Sistema de Informação sobre Mortalidade* - SIM), Primary Care Information System (*Sistema de Informação da Atenção Básica* - SIAB), Notifiable Diseases Information System (*Sistema de Informação de Agravos de Notificação* - SINAN), Hospital Information System of the Unified Health System (*Sistema de Informações Hospitalares do Sistema Único de Saúde* - SIH/SUS), National Registry of Health Establishments (*Cadastro Nacional de Estabelecimentos de Saúde* - CNES), and data from National Supplementary Health Agency (*Agência Nacional de Saúde Suplementar* - ANS) and the Brazilian Institute of Geography and Statistics (*Instituto Brasileiro de Geografia e Estatística -* IBGE).

**Figure 1.**
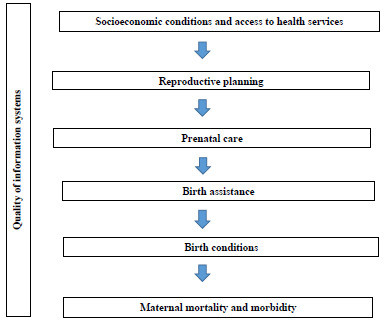
Theoretical model of the determinants of maternal mortality.

The first step in product development was the development of a theoretical model of the determinants of maternal death, based on available scientific literature^6^ (Figure 1). Using this model as a reference, all sources of information that made data available by Brazilian municipality, the lowest level of geographic aggregation used, were explored. Considering the theoretical model and available data, a list of potential indicators was made, which were calculated by federation unit, using the municipality of Rio de Janeiro as a reference to validate the calculation method. This choice was due to the good health surveillance system of the Municipal Health Secretariat of Rio de Janeiro (*Secretaria Municipal de Saúde do Rio de Janeiro* - SMS/RJ), as well as the participation of a professional from SMS/RJ in the research team, which facilitated access to municipal databases, whenever there were doubts.

After refining the initial list, the indicators included in the panel were selected, summarized in Chart 1. All indicators were calculated for the period 2012-2020, by Brazilian municipality. ETL (data extraction, transformation, and loading) routines were developed for the selected indicators, with the data stored in Elasticsearch. Information sources used are described in Chart 2.

**Chart 1.** Indicators available on the “Maternal health surveillance” panel, with respective calculation method, source of information, and reference value.

Indicator	Calculation method	Source of information	Reference value
Block 1 - Socioeconomic conditions and access to health services			
MHDI	Dimensions of the Global HDI (longevity, education, and income), adapting the methodology to the Brazilian context and the availability of national indicators.	Atlas Brasil	0.00-0.499=very low; 0.500-0.599=low; 0.600-0.699=medium; 0.700-0.799=high; 0.800-1.00=very high^18^
Percentage of LB according to mother’s age range	Number of LB by maternal age divided by total LB and multiplied by 100.	SINASC	National average
Percentage of LB according to mother’s race/color	Number of LB by maternal skin color divided by total LB and multiplied by 100.	SINASC	National average
Percentage of LB according to mother’s education	Number of LB by maternal education divided by total LB and multiplied by 100.	SINASC	National average
Percentage of women aged 10 to 49 years who are exclusive users of SUS	(Female population aged 10 to 49 years minus female population aged 10 to 49 years benefiting from medical insurance) divided by the female population aged 10 to 49 years and multiplied by 100.	ANS, IBGE	National average
Population coverage with family health teams	Annual average of the population assisted by family health teams divided by the total population of the municipality and multiplied by 100.	SIAB	95% (SDG)^*^ ^11^
Block 2 - Reproductive planning			
Specific fertility rate in women under 20 years of age	Number of LB of women under 20 years old divided by the female population aged 10 to 19 years old and multiplied by 1,000.	SINASC, IBGE	<30 per 1,000 (developed countries)^15^
Percentage of multiparous women	Number of LB of women with >3 previous births divided by total LB and multiplied by 100.	SINASC	National average
Unsafe abortion rate per 1,000 women of childbearing age^19^	Number of hospitalizations for abortion in public and private services multiplied by the correction factor for hospitalizations for spontaneous abortion and for induced abortions without hospital admission divided by the female population aged 10 to 49 years and multiplied by 1,000.	SIH/SUS, ANS, IBGE	National average
Unsafe abortion ratio per 100 LB^19^	Number of hospitalizations for abortion in public and private services multiplied by the correction factor for hospitalizations for spontaneous abortion and for induced abortions without hospital admission divided by total LB and multiplied by 100.	SIH/SUS, ANS, SINASC	National average
Block 3 - Prenatal care			
Prenatal care coverage	Number of LB of mothers with any prenatal consultation divided by total LB and multiplied by 100.	SINASC	95% (WHO recommendation)^†^ ^9^
Percentage of women starting early prenatal care	Number of LB of mothers who started prenatal care in the first trimester of pregnancy divided by total LB and multiplied by 100.	SINASC	95% (WHO recommendation)^†^ ^9^
Percentage of women with more than seven prenatal consultations	Number of LB of mothers with >7 prenatal consultations divided by total LB and multiplied by 100.	SINASC	95% (WHO recommendation)^†^ ^9^ ^,^ ^12^
CS incidence rate	Number of CS cases in <1 year divided by total LB and multiplied by 1,000.	SINAN, SINASC	≤0.5 per 1 thousand LB (international goal)^9^
Block 4 - Delivery assistance			
Percentage of births by cesarean section	Number of LB per cesarean section divided by total LB and multiplied by 100.	SINASC	10-15% (WHO reference)^13^
Percentage of births according to Robson group	Number of LB per Robson group divided by total LB and multiplied by 100.	SINASC	National average
Percentage of births by cesarean section in each Robson group	Number of LB by cesarean section in each Robson group divided by total LB in each Robson group and multiplied by 100.	SINASC	Group 1=10%; Group 2=20 to 35%; Group 3=3%; Group 4=15%; Group 5=50 to 60%; Group 10=30% (WHO reference)^14^; Groups 6 to 9=national average.
Relative contribution of each Robson group to the total cesarean section rate	Number of LB by cesarean section in each Robson group divided by total LB per cesarean section and multiplied by 100.	SINASC	National average
Percentage of births according to place of occurrence	Number of LB per birth location divided by total LB and multiplied by 100.	SINASC, CNES	National average of births occurring in the municipality of residence
Median travel for births occurring outside the mother's municipality of residence according to complexity of the birth care service	Median travel in kilometers between the geographic coordinate of the urban perimeter of the mother’s municipality of residence and that of the municipality in which the birth occurred. Complexity of the service defined according to adult ICU bed availability registered in CNES.	SINASC, CNES	No reference standard^‡^
Block 5 - Birth conditions			
Percentage of births with low birth weight	Number of LB with birth weight <2,500 g divided by total LB and multiplied by 100.	SINASC	30% reduction by 2025 in relation to values observed in 2006-2010 (international goal)^10^
Percentage of preterm births	Number of LB with GA <37 weeks divided by total LB and multiplied by 100.	SINASC	10% (developed countries)^16^
Percentage of early term births	Number of LB with GA 37 or 38 weeks divided by the total LB and multiplied by 100.	SINASC	20% (developed countries with a low percentage of cesarean births)^17^
Block 6 - Maternal mortality and morbidity			
Number of maternal deaths	Total number of maternal deaths after investigation.	SIM	No reference standard^§^
MMR	Number of maternal deaths divided by total LB multiplied by 100,000.	SIM, SINASC	<30 per 100,000 live births (Brazil SDG)^11^
Percentage of maternal deaths due to direct obstetric causes	Number of maternal deaths from direct obstetric causes divided by the total number of maternal deaths multiplied by 100.	SIM	National average
Percentage of direct maternal deaths due to specific causes	Number of maternal deaths from specific direct obstetric causes (abortion, hypertension, hemorrhage, and infections) divided by the total number of maternal deaths from direct obstetric causes multiplied by 100.	SIM	National average
Percentage of obstetric hospitalizations classified as severe maternal morbidity	Number of SMM cases divided by the total number of public obstetric hospitalizations multiplied by 100	SIH/SUS	National average
Percentage of cases of severe maternal morbidity according to specific causes	Number of SMM cases according to cause (hypertension, hemorrhage, infection) divided by the total number of SMM cases in public obstetric hospitalizations multiplied by 100.	SIH/SUS	National average
Percentage of SMM cases according to management indicators	Number of SMM cases according to management indicator (transfusion, surgery, ICU admission, ALS >7 days) divided by the total of SMM cases in public obstetric hospitalizations multiplied by 100.	SIH/SUS	National average
Quality of information			
SINASC Coverage	Number of LB in SINASC divided by the estimated number of births multiplied by 100.	IBGE	90%^7^
SIM coverage	Number of deaths in SIM divided by the estimated number of deaths multiplied by 100.	IBGE	90%^7^
Percentage of deaths of women of childbearing age investigated	Number of WCA deaths investigated divided by total WCA deaths and multiplied by 100.	Tabnet SIM	90% (national goal)^20^
Percentage of maternal deaths investigated	Number of maternal deaths investigated divided by the total number of maternal deaths and multiplied by 100.	Tabnet SIM	100% (national goal)^20^
Degree of incompleteness	Number of variables with blank or missing records divided by total LB and multiplied by 100.	SINASC	<5%^8^

MHDI: Municipal Human Development Index; HDI: Human Development Index; LB: live births; SINASC: Live Birth Information System (*Sistema de Informação sobre Nascidos Vivos*); ANS: National Supplementary Health Agency (*Agência Nacional de Saúde Suplementar*); IBGE: Brazilian Institute of Geography and Statistics (*Instituto Brasileiro de Geografia e Estatística*); SIAB: Primary Care Information System (*Sistema de Informação da Atenção Básica*); SIH/SUS: Hospital Information System of the Unified Health System (*Sistema de Informações Hospitalares do Sistema Único de Saúde*); WHO: World Health Organization; CS: congenital syphilis; SINAN: Notifiable Diseases Information System (*Sistema de Informação de Agravos de Notificação*); GA: gestational age; CNES: National Registry of Health Establishments (*Cadastro Nacional de Estabelecimentos de Saúde*); SIM: Mortality Information System (*Sistema de Informação sobre Mortalidade*); MMR: Maternal Mortality Ratio; SDG: Sustainable Development Goals; SMM: severe maternal morbidity; ICU: Intensive Care Unit; ALS: average length of stay;

WCA: woman of childbearing age.

*The SDG’ aim is universal coverage of the population with basic care. We chose the reference value of 95% due to the possibility of error in population estimates. In municipalities with high health insurance coverage, coverage with family health teams must cover at least the percentage of the population that depends exclusively on SUS; ^†^95% is the value recommended by the WHO for process indicators to prevent vertical transmission of syphilis and HIV. The same value was adopted for prenatal indicators; ^‡^due to the great Brazilian diversity, we chose not to define a national reference value. In general, the higher the median displacement, the greater the probability of delay in delivery care; ^§^reference value not applicable.

**Chart 2.** Access to information sources used in the development of the “Maternal health surveillance” panel.

Database	Period	Access
Atlas Brasil (IDHM)	2010	http://www.atlasbrasil.org.br/ranking
SINASC microdata	2012-2020	https://pcdas.icict.fiocruz.br/ (access via registration and access authorization)
SIM-DOMAT microdata	2012-2020	https://github.com/rfsaldanha/microdatasus
SIH/SUS microdata	2012-2020	https://github.com/rfsaldanha/microdatasus
CNES	2012-2020	https://pcdas.icict.fiocruz.br/ (access via registration and access authorization)
SIAB	2012-2020	https://egestorab.saude.gov.br/paginas/acessoPublico/relatorios/relHistoricoCoberturaAB.xhtml
SINAN	2012-2020	http://indicadoressifilis.aids.gov.br
Tabnet DataSUS (population estimates)	2012-2020	http://tabnet.datasus.gov.br/cgi/deftohtm.exe?ibge/cnv/popsvsbr.def
Tabnet ANS (health plan beneficiaries)	2012-2020	www.ans.gov.br/anstabnet/cgi-bin/dh?dados/tabnet_02.def
Hospital admission database in supplementary health	2015-2020	https://dados.gov.br/dados/conjuntos-dados/procedimentos-hospitalares-por-uf
Distance and road travel time matrices between Brazilian municipalities (CEDEPLAR/UFMG)^21^	2020	https://www.dropbox.com/sh/1gx8xwdddrwz2gt/AADzJoAeDD7KXTKZoqPLWiJza?dl=0
Tabnet SIM (death investigation coverage)	2012-2020	http://tabnet.datasus.gov.br/cgi/deftohtm.exe?sim/cnv/mat10br.def
SIM coverage	2015-2020	https://www.ibge.gov.br/estatisticas/sociais/populacao/26176-estimativa-do-sub-registro.html?edicao=32265&t=resultados
SINASC Coverage	2015-2020	https://www.ibge.gov.br/estatisticas/sociais/populacao/26176-estimativa-do-sub-registro.html?edicao=32265&t=resultados

MHDI: Municipal Human Development Index; SINASC: Live Birth Information System (*Sistema de Informação sobre Nascidos Vivos*); SIM-DOMAT: Maternal Death Mortality Information System (*Sistema de Informação sobre Mortalidade óbitos maternos*); SIH/SUS: Hospital Information System of the Unified Health System (*Sistema de Informações Hospitalares do Sistema Único de Saúde*); CNES: National Registry of Health Establishments (*Cadastro Nacional de Estabelecimentos de Saúde*); SIAB: Primary Care Information System (*Sistema de Informação da Atenção Básica*); SINAN: Notifiable Diseases Information System (*Sistema de Informação de Agravos de Notificação*); ANS: National Supplementary Health Agency (*Agência Nacional de Saúde Suplementar*); CEDEPLAR/UFMG: Center for Development and Regional Planning at Universidade Federal de Minas Gerais (*Centro de Desenvolvimento e Planejamento Regional da Universidade Federal de Minas Gerais*); PCDaS: Data Science Platform applied to Health/Fiocruz (*Plataforma de Ciência de Dados aplicada à Saúde/Fiocruz*).

The last step was the development of the online interactive panel, built using the Shiny package of R language^22^. Various visualization possibilities were tested, as well as explanatory texts developed for each indicator (definition, calculation method, quality assessment, how to interpret). The panel allows data to be viewed according to different geographic areas (municipality, state micro and macro regions, federation unit, macro regions of the country, and country), using the files available in DATASUS to define health sectors^23^.

For all indicators, coverage and information completeness data are presented to alert users about possible errors in the indicator, in the case of low coverage^7^ or high incompleteness^8^, and the need for the result to be interpreted with caution. When data for this assessment are not available, an alert is made about errors in the information system that could result in limitations in the use of the indicator. The message described in the alert aims to raise awareness among managers of the importance of actions to continuously improve information systems.

Reference standards are presented for all panel indicators, which allow the evaluation performance in each selection made. The following were used as reference:

1. International goals^9^ ^,^ ^10^;
2. National goals^11^;
3. Recommendations from the World Health Organization (WHO)^9^ ^,^ ^12^ ^,^ ^13^ ^,^ ^14^;
4. Results observed in other countries^15^ ^,^ ^16^ ^,^ ^17^;
5. National average of the indicator (Chart 1).

The indicators are presented at three levels. In the first, all indicators for the selected geographic area and year are presented, with a report available for printing. In the second, the historical series of indicators for each block of the theoretical model is presented, making it possible to select the period to be analyzed, as well as comparators (for example, other municipalities in the same micro-region or with a similar Municipal Human Development Index - MHDI^18^). In the third, each indicator is presented individually, with its documentation and other visualization possibilities. Documentation of the calculation method for all indicators is available in the panel menu, under the “indicator documentation” icon (https://observatorioobstetrico.shinyapps.io/painel-vigilancia-saude-materna/).

The panel also has the access link to “Aparecida: a story about the vulnerability of Brazilian women to maternal death.” It is an educational material, based on the story of a fictional woman, but which reflects the story of many Brazilian women. It begins when Aparecida has an unplanned pregnancy at age 15 and ends when she experiences severe maternal morbidity during the birth of her fifth child. Throughout the story, situations are presented that reflect Aparecida’s vulnerability to maternal death, which can be captured by the indicators presented in the panel. At each stage of the story, users are offered the opportunity to “find out more”, in which national and/or international data on the indicators covered are presented, aiming to assist in understanding the determinants of maternal death and in the interpretation and possibility of using the indicators displayed on the panel. At the end of “to find out more”, a link to access the indicator panel is always available, integrating the two products.

### Relevance of health indicators available on the panel

In Block 1, “Socioeconomic conditions and access to health services”, five indicators are presented. MHDI is a composite measure that uses the same dimensions as Global HDI (longevity, education, and income), adapting the global methodology to the Brazilian context and the availability of national indicators^18^. It was used due to its inverse relationship between HDI and MMR^24^: the lower the HDI, the higher the MMR. MHDI cannot be compared to the HDI of other countries, but it allows comparison between Brazilian municipalities, identifying those with lower values and, therefore, greater vulnerability to maternal death. In Brazil, higher MMR values are observed in women at extreme ages, in women with less education and in those with black or indigenous skin color/race^5^. The indicator that presents the distribution of LB according to age, education and mother’s skin color aims to identify the municipalities with the highest percentage of most vulnerable women. The indicator “percentage of women aged 10 to 49 who are exclusive users of SUS” was calculated based on ANS data relating to beneficiaries of medical health plans. This indicator reflects the social vulnerability of women, since, in Brazil, access to health plans is associated with a better economic situation^25^ ^,^ ^26^, and indicates the percentage of women aged 10 to 49 years who depend exclusively on SUS, contributing to the planning of health actions and services. Finally, population coverage with Family Health Teams is relevant, as it is the gateway to the health system, with health promotion actions, chronic disease control, and reproductive planning and PN assistance services.

In Block 2, “Reproductive Planning”, four indicators are presented that indirectly reflect access to reproductive planning services, as no Brazilian information system contains population information on the use of contraceptive methods. The fertility rate in women under 20 years of age was chosen because it presents high values in Latin America, with its reduction being one of the goals of the Pan American Health Organization (PAHO) for the year 2030^27^. Pregnancy in women under 20 years of age occurs with frequency in an unplanned manner, being associated with limited educational and work opportunities, perpetuating intergenerational cycles of poverty^27^. The indicator “percentage of multiparous women” was chosen because it indicates possible barriers to reproductive planning, in a context of decreasing fertility rates in the country^15^. Despite the low values, a higher proportion than the national average may indicate local barriers that should be investigated. Finally, the indicators “rate of unsafe abortions per 1,000 women of childbearing age (WCA)” and “ratio of unsafe abortions per 100 LB” estimate the frequency of unsafe abortions (UA) in WCA and the UA/LB ratio, respectively. The higher the value, the greater the frequency of UA and unmet contraceptive needs. For the calculation, the methodology by Guttmacher Institute^19^ was used, which proposes the application of correction factors (for hospitalizations due to spontaneous abortion and terminations of pregnancy that did not result in hospital admission) to the number of hospitalizations due to abortion, to estimate the total number of UA.

In Block 3, “Prenatal care”, there are four indicators. Brazil has almost universal coverage of PN assistance^28^, and a higher proportion of women without at least one PN consultation is an indicator of social vulnerability and possible barriers to accessing primary care. Early initiation of PN is essential for adequate care during pregnancy, with values still low in the country. In the “Birth in Brazil” study, carried out in 2011/2012, only a quarter of women started PN early, with late start associated with difficulties in diagnosing pregnancy, personal issues, and access barriers^28^. For the number of PN consultations, the WHO recommendations were considered, which since 2016 has indicated a minimum number of eight PN consultations for a pregnant woman at usual risk^12^. In the absence of information that allows the assessment of the content of PN care, we used the incidence of congenital syphilis as a marker of the quality of this care, as it is an outcome to be avoided exclusively with control actions carried out during pregnancy^9^.

In Block 4, “Childbirth assistance”, two sets of indicators are presented: indicators related to the percentage of births by cesarean section and indicators that reflect the displacement of women to birth assistance. There is no evidence that a population cesarean rate higher than 15% is associated with lower MMR^29^ ^,^ ^30^, but, in Brazil, cesarean sections have been the main type of birth since 2009, with a rate higher than 50%^31^. The WHO recommends the use of Robson groups to analyze cesarean sections^14^. In this methodology^32^, women are classified into ten groups, and the size of the groups, the cesarean rate in each group and the proportional contribution of each group to the overall cesarean rate are evaluated. The larger the group size and the higher the cesarean section rate in the group, the greater its contribution to the global cesarean rate, with groups 2 and 5 being the most relevant in Brazil^33^. The second group of indicators is based on the model of three delays related to maternal death^34^, in which the longer the delay in receiving appropriate care, the greater the risk of maternal death^35^. With the data available in information systems, it is possible to evaluate the second delay, *i.e*., the delay in accessing a birth care service^34^. The panel presents the percentage of births according to place of occurrence and the median travel distance to birth care services located outside the municipality of residence, according to the level of complexity of the service. In general, the greater the percentage of births outside the municipality of residence and the greater the median displacement, the greater the woman’s vulnerability to maternal death and the greater the need for bed regulation and safe transportation, especially for women with high gestational risk.

In Block 5, “Birth conditions”, indicators related to the newborn are presented, but which reflect the quality of PN and childbirth care. The percentage of births with low birth weight (weight <2,500 g)^36^ and preterm births (gestational age <37 weeks)^16^ are the main risk factors for infant mortality and can be reduced with actions developed during PN care, such as treating complications and reducing risk factors like smoking and alcohol and drug use. Early term births (born at 37 to 38 weeks) present a higher risk of complications than those born at full term (with 39 and 40 gestational weeks)^37^, with a higher percentage of early term births being observed in places with a higher percentage of cesarean sections^38^.

Finally, Block 6 presents indicators of “Maternal mortality and morbidity.” Generally, in places with a small number of deaths, MMR is not calculated, due to the large fluctuation of the indicator, and only the absolute number of deaths is presented. However, we chose to present both indicators, even in small municipalities, as we understand that the high MMR, even with the occurrence of just one death, demonstrates the severity of the indicator, which could be relativized by the low frequency of the outcome. The percentage of deaths due to direct maternal causes, which are most affected by the quality of PN and childbirth care, is also presented, as well as the main specific causes of these deaths^39^. For maternal morbidity, the severe maternal morbidity indicator (SMM) is presented and calculated from SIH/SUS data, according to the WHO classification for “potentially life-threatening conditions”^40^ ^,^ ^41^. In addition to the percentage of obstetric hospitalizations classified as SMM, the present study brings the main causes of morbidity (hypertension, hemorrhage, and infections) as well as the main management indicators (admission to the Intensive Care Unit - ICU, hospitalization for more than 7 days postpartum, transfusion of blood products and surgical procedures).

### Implications for epidemiological surveillance

The “Maternal health surveillance” panel uses data that are already available in several Brazilian information systems. Its innovation consists of integrating these data into a single digital platform, providing indicators calculated for different periods and geographic areas, allowing comparisons between locations and reference standards. The panel also provides several informative texts that aim to democratize access and use of health indicators by managers, health professionals, researchers, students and social movements. It should be noted that the analysis of all indicators must consider the context of the period analyzed. For example, the shortage of penicillin in the country, in the period 2013-2017, affected the incidence of cases of congenital syphilis^42^, while the Covid-19 pandemic increased the number of maternal deaths^43^.

The material “Aparecida: a story about the vulnerability of Brazilian women to maternal death”, integrated into the indicator panel, complements information on health indicators, promoting knowledge about the determinants of maternal death and its preventability. Maternal mortality is an indicator that reflects the situation of women in society, and the reduction of maternal deaths and the promotion of women’s health depend not only on health services, but on intersectoral policies and actions^2^ ^,^ ^6^. We believe that its use can be encouraged among maternal mortality committees and health councils, encouraging their work to be more autonomous and, at the same time, technically and scientifically based.

We understand that the use of the indicator panel has the potential to expand maternal health surveillance, especially in the more than 4 thousand Brazilian municipalities that have a small population and do not record maternal deaths regularly, but also in those that report maternal deaths more frequently. The investigation of maternal deaths, which has not yet reached the expected national target of 100% coverage, aims at an in-depth analysis of death cases^39^, while the data made available in the panel allow us to verify to what extent a situation identified in the death also affects other women from that same municipality, making them vulnerable to a negative outcome. Furthermore, the low frequency of deaths may prevent all existing vulnerability situations from appearing in the deaths investigated. They are, therefore, strategies that complement each other.

The panel presents indicators that, although calculated from data available in information systems, require detailed calculations and are not readily available for consultation. Among them, we highlight the percentage of women aged 10 to 49 years who are exclusive users of SUS, the fertility rate in women under 20 years of age, the rate of UA per 1,000 WCA, the ratio of UA per 100 LB, the proportion of births according to place of occurrence, the median travel distance to the birth care service and indicators related to SMM. All these indicators aggregate relevant information about women’s health, and their incorporation into epidemiological surveillance is an important contribution of this tool. Some indicators, such as UA rate, UA ratio, and SMM indicators, present methodological challenges and could be improved through their use and review by other researchers.

Specifically, MMG indicators represent an advance in maternal morbidity surveillance. Since 2011, the WHO has recommended the analysis of SMM as a complementary strategy to the study of maternal death^40^, as it is more frequent and presents the same determinants, allowing for more robust analyses^41^. The SMM indicator calculated based on the WHO criteria for “potentially life-threatening conditions” is presented in the panel, representing serious morbidities. The availability of the frequency of these cases, as well as their causes and main management indicators, represents an advance in the epidemiological surveillance of maternal morbidity and can help managers, especially in municipalities that do not record maternal deaths regularly, to plan their health services. It should be noted, however, that these cases only refer to SMM identified in public hospitalizations, with limited information in municipalities with a high percentage of women benefiting from health plans.

The panel also encourages the improvement of information quality, by presenting quality data integrated with the indicators available, drawing attention to the need for constant improvement in the coverage of information systems and the quality of data recording.

The biggest challenge in developing the panel was the use of a large number of databases from information systems with variable implementation time, purpose, coverage, and filling quality. The development of the project showed the importance of multidisciplinary teamwork, involving data scientists, statisticians, epidemiologists, professionals who work in health surveillance and experts on the topic. It also showed the importance of studying all available documentation about the information system that will be used, avoiding errors resulting from misunderstanding of its variables and way of functioning; as well as the need to explore the database to identify inconsistencies and variations in the filling pattern that may reflect regional differences and not necessarily filling errors, especially in a continental country like Brazil.

To conclude, the products presented have the potential to expand epidemiological surveillance of maternal health and its determinants, contributing to the formulation of health policies and actions that promote women’s health and reduce maternal mortality.

As future developments, we identified the need for periodic updating of the panel, with its planned biannual update. The panel has a flexible architecture that allows, whenever necessary, the inclusion of new indicators, and its expansion is already underway to include “maternal and perinatal health surveillance.” There is also the possibility of developing an intra-municipal panel, with disaggregated indicators, which would be very relevant for larger municipalities in which the municipal average can hide intra-municipal inequalities.
